# The queen’s gut refines with age: longevity phenotypes in a social insect model

**DOI:** 10.1186/s40168-018-0489-1

**Published:** 2018-06-18

**Authors:** Kirk E. Anderson, Vincent A. Ricigliano, Brendon M. Mott, Duan C. Copeland, Amy S. Floyd, Patrick Maes

**Affiliations:** 1 USDA-ARS Carl Hayden Bee Research Center, 2000 E. Allen Rd, Tucson, AZ 85719 USA; 2 Department of Microbiology, School of Animal & Comparative Biomedical Sciences, University of Arizona, Tucson, AZ 85721 USA; 3 Department of Entomology and Center for Insect Science, University of Arizona, Tucson, AZ 85721 USA

**Keywords:** Honey bees, Acetobacteracaeae, *Bifidobacterium*, *Parasaccharibacter apium*, *Lactobacillus kunkeei*, Ileum, Aging, Core microbiota, Bacteria, Oxidative stress

## Abstract

**Background:**

In social insects, identical genotypes can show extreme lifespan variation providing a unique perspective on age-associated microbial succession. In honey bees, short- and long-lived host phenotypes are polarized by a suite of age-associated factors including hormones, nutrition, immune senescence, and oxidative stress. Similar to other model organisms, the aging gut microbiota of short-lived (worker) honey bees accrue Proteobacteria and are depleted of *Lactobacillus* and *Bifidobacterium,* consistent with a suite of host senescence markers. In contrast, long-lived (queen) honey bees maintain youthful cellular function with much lower expression of oxidative stress genes, suggesting a very different host environment for age-associated microbial succession.

**Results:**

We sequenced the microbiota of 63 honey bee queens exploring two chronological ages and four alimentary tract niches. To control for genetic and environmental variation, we quantified carbonyl accumulation in queen fat body tissue as a proxy for biological aging. We compared our results to the age-specific microbial succession of worker guts. Accounting for queen source variation, two or more bacterial species per niche differed significantly by queen age. Biological aging in queens was correlated with microbiota composition highlighting the relationship of microbiota with oxidative stress. Queens and workers shared many major gut bacterial species, but differ markedly in community structure and age succession. In stark contrast to aging workers, carbonyl accumulation in queens was significantly associated with increased *Lactobacillus* and *Bifidobacterium* and depletion of various Proteobacteria.

**Conclusions:**

We present a model system linking changes in gut microbiota to diet and longevity, two of the most confounding variables in human microbiota research. The pattern of age-associated succession in the queen microbiota is largely the reverse of that demonstrated for workers. The guts of short-lived worker phenotypes are progressively dominated by three major Proteobacteria, but these same species were sparse or significantly depleted in long-lived queen phenotypes. More broadly, age-related changes in the honey bee microbiota reflect the regulatory anatomy of reproductive host metabolism. Our synthesis suggests that the evolution of colony-level reproductive physiology formed the context for host-microbial interactions and age-related succession of honey bee microbiota.

**Electronic supplementary material:**

The online version of this article (10.1186/s40168-018-0489-1) contains supplementary material, which is available to authorized users.

## Background

Honey bees (*Apis mellifera*) function as a cooperating group of individuals (colonies) characterized by division of labor [1]. Reproduction is performed by long-lived queen phenotypes while short-lived workers perform a variety of nutrient processing and other tasks that support the reproductive effort [2]. Both longevity phenotypes can result from identical genomes, but queens can live over ten times as long as workers and consume a very different diet [3]. Beginning as newly hatched larvae, queen vs. worker (caste) development is controlled by signaling molecules found in different diets. Pollen exposure halts queen development while royal jelly promotes queen development [4, 5]. Nurse workers gorge on pollen to synthesize royal jelly fed to queens throughout their lives. Royal jelly is functionally analogous to mammalian breast milk comprised of a complete diet and antioxidant, antimicrobial, and immunoregulatory properties [6, 7]. Attributed to caste-specific diets, the phospholipid profile of aging workers becomes increasingly susceptible to oxidative stress, but the queen profile remains stable with age [8]. Consistent with these results, antioxidant gene expression increases in aging workers but not queens [9, 10]. Workers live longer when fed the queen diet (royal jelly) as compared to a pollen diet [11]. Collectively, these results suggest that the drastically different lifespans and diets associated with division of labor in honey bees provide a model for mechanisms of diet, aging, and microbiota [12, 13].

Division of labor in social insects is organized around nutrition and reproduction. In honey bees, this social organization is attributed to the evolutionary repurposing of an egg yolk glyco-lipoprotein (vitellogenin) to serve as nutritional currency throughout the colony [14]. The oldest honey bees leave the hive to forage for nectar, pollen, and water. Collected pollen is converted by young workers into two major forms of nutritional currency, one internal, vitellogenin, expressed mostly by abdominal fat body, and one external, royal jelly, shared as social currency among nestmates. In workers, much of the vitellogenin released into the hemolymph is diverted to worker head glands to produce royal jelly [15]. Royal jelly secretions from young (nurse) bees are fed via oral trophallaxis to growing larvae and the queen. In turn, much of the royal jelly fed to queens is converted internally to vitellogenin, to support massive egg production [14]. Vitellogenin is expressed constitutively throughout the queens’ internal anatomy [9, 16]. Like royal jelly, vitellogenin is a multipurpose superfood that functions in immunity, detoxification, oxidative stress, nutrition, and longevity [9, 16–18]. Older foragers no longer produce jelly, but often beg for and receive small doses from younger nurse bees.

Reproductive division of labor underlies changes in microbiota composition both proximately and ultimately [19, 20]. Workers feeding queen vs. worker-destined larvae differ markedly for antimicrobial gene expression associated with royal jelly production in their head glands [21]. Following emergence as winged adults, queen and worker guts are colonized by very different microbiota [20, 22, 23]. Although highly antimicrobial, the queen’s diet of royal jelly enhances the growth in vitro of at least two bacterial species associated with the queen microbiota [7]. Accordingly, the worker phenotype is affected by pollen consumption that occurs concurrent with early adult succession of gut microbiota [24]. Experiments with conventionalized honey bee workers and pollen consumption indicate that bacterial fermentation products produced during the digestion of recalcitrant pollen shells influence host insulin signaling and the production of vitellogenin [25]. Vitellogenin and life expectancy decrease dramatically as workers transition to foraging [8] and the hindgut microbiota shifts with age.

In worker hindguts, fermentation products of gut bacteria are produced according to microbiota structure [25]. A variety of environmental insults can perturb microbiota structure (dysbiosis), altering immune expression, producing oxidative damage and host inflammation [25–28]. Dysbiotic workers suffer developmental deficiencies and early mortality suggesting that the suppression of oxidative stress via microbiota maintenance is critical for gut health and host longevity [25, 29]. Similar to gut dysbiosis in response to early life insult, age-associated succession of gut microbiota in worker bees shows increased Proteobacteria with relative decreases in core *Bifidobacterium* and *Lactobacillus*, the same general results found in many other microbiota models including insects and mammals [12, 30]. Unlike workers, the queen does not show greater antioxidant expression with age suggesting that antioxidant function is performed differently or managed by her diet [9]. Vitellogenin hemolymph concentration, constant royal jelly ingestion, and perhaps the microbiota contribute to antioxidant function in long-lived queens.

While research on the worker microbiota has progressed rapidly, little is known of queens. Based on a small sample size and high-throughput 16S subunit of the ribosomal RNA gene (16S rRNA) gene sequencing from whole guts, the queen and worker microbiota differ in taxonomic membership and community structure [19, 20, 31]. Unlike workers, the early queen gut seems dominated by two distinct species of Acetobacteraceae, *Parasaccharibacter apium* and an unnamed group referred to as “Alpha 2.1” [12]. Alpha 2.1 is prevalent in guts of older workers, but *P. apium* occupies a variety of nutrition-rich niches associated with honey bees and thrives in the presence of royal jelly [7, 32, 33]. Capable of gut colonization, *P. apium* is correlated with disease agents in adult bumblebees [34] and, in honey bees, implicated in poor worker health, increased mortality, worker gut dysbiosis, and strain-dependent effects on larval and pupal survival [12, 29, 33]. Often occurring with *P. apium*, *Lactobacillus kunkeei* is prevalent/abundant in queens, also considered a probiotic, and associated with worker disease and dysbiosis [12, 35]. *L. kunkeei* has been designated as a hive (not a gut) bacterium due to its association with fructose-rich niches like honey and honey-rich pollen storage [32, 36]. Thus, investigations of honey bee microbiota require a careful consideration of social and functional context including host longevity, caste specificity, developmental stage, potential refugia, and transmission from nutrition-related niches [22, 23, 37].

Here we test the hypothesis that lifespan differences in a social insect model are associated with age-based microbial succession. We sample known age queens from different genetic and environmental backgrounds and compare our findings to the extensive preexisting characterization of known age workers. We define the aging queen microbiota by deep sequencing the 16S rRNA gene from four alimentary tract niches that differ in many ways including physiological function, pH, and oxygen exposure. To accompany each amplicon library, we normalize bacterial cell counts with qPCR and rRNA gene copy number. Finally, we quantify protein oxidation in the fat body tissue of each queen to test the hypothesis that biological age differs from chronological age and that the accrual of oxidation products in aging queens is associated with differences in the microbiota.

## Methods

### Queen sampling

Our sampling design distinguished environmental exposure from chronological age. We sampled four different sets of queens: young queens (first year, *n* = 31) aged 4–6 months and old queens (second year, *n* = 32) aged 16–18 months. To control for source variation, we sampled old and young queens from similar and different backgrounds. The primary multivariate analysis of variance (MANOVA) model contains two main effects and an interaction effect, asking whether the variation in queen microbiota depends on age, background, or an interaction of both factors.

We sampled a total of 63 queens. Half (*n* = 32) of these queens were sourced from a large migratory beekeeping operation based in southern California. Referred to as the “CA” source, these Italian queens (*Apis mellifera ligustica*) were purchased from the same queen breeder in different years (mid-March of 2015 and 2016). Both sets of queens were sampled in mid July 2016. Thus, “older” CA queens (*n* = 16) were sampled from colonies that had survived 16.5 months and experienced almond pollination and two seasons of alfalfa pollination in the Imperial valley of southern California. Following almond pollination in 2016, “young” CA queens (*n* = 16) were introduced via colony splits in March, experienced one season of alfalfa pollination, and were sampled at 4.5 months of age.

The other half of our sampled queens, referred to as “AZ” queens (*n* = 31) were sourced from two very different environmental and genetic backgrounds. We sampled “young” AZ queens (*n* = 15) from the Carl Hayden Bee Research Center in Tucson, Arizona. Delivered and installed with 3000 young worker bees (package bees) in early May 2016, these Italian queens (*Apis mellifera ligustica*) were exposed to varied pollen and nectar sources typical of the Sonoran desert, but not intensive agriculture or bulk transportation events. Young AZ queens were sampled in early October 2016 at 5.7 months of age. In contrast, old AZ queens originated from a Northern migratory beekeeping operation that raises *Apis mellifera carnica* queens*.* These queens were introduced in April of 2015 to colony splits in the foothills east of Turlock, California following almond pollination. Colonies then experienced the summer in North Dakota making honey, pollinating oilseed crops, sunflowers, and canola. Colonies then overwintered in a temperature-controlled warehouse in Idaho (November–January) and pollinated almonds in central California (February). The colonies were then delivered to Tucson, Arizona in March of 2016 where they flourished for 7 months before queens were sampled in early October at 18 months of age.

All 63 queens were collected into sterile 2.0-ml tubes and immediately frozen on dry ice and stored at − 20 °C for DNA extraction. Queens were dissected under sterile conditions. Four tissue types were extracted to typify the queen microbiota: mouth parts, midgut, ileum, and rectum. Mouthparts were unfolded out of the head capsule and detached proximal to the labrum with sterile scissors. Individuals were then pinned through the thorax and the digestive tract was accessed by removing the dorsal abdominal sclerites. The entire digestive tract was removed and floated in 70% EtOH to wash and separate the midgut, ileum, and rectum. The abdominal fat body and attached dorsal sclerites were retained as a single unit to quantify biological age.

### Queen aging assay

As a proxy for biological age, we quantified molecular by-products that cannot be excreted, but accumulate with age in abdominal fat body tissues. In honey bees, the accumulation of oxidized proteins (carbonyl groups) in the fat body is recognized as a marker of chronological age [38]. Carbonyl content of total fat body protein homogenates was determined using a Protein Carbonyl Content Assay Kit (MAK094; Sigma-Aldrich). Briefly, whole fat bodies were homogenized in 600 μl of 1X TE buffer. The supernatant was treated with a final concentration of 10 mg/ml streptozotocin to precipitate nucleic acids. The supernatant was decanted then reacted with 2,4-dinitrophenylhydrazine (DNPH) to form stable dinitrophenyl hydrozone adducts. Derivatized proteins were precipitated with trichloroacetic acid and were washed three times with acetone. The samples were resuspended in 100 μl of 6 M guanidine (pH 2.3). Protein oxidation, expressed as nanomoles of carbonyl groups per milligram of protein, was calculated by absorbance at 345 nm relative to the millimolar extinction coefficient of aliphatic hydrozones (22 mM^−1^ cm^−1^). The protein content of each sample was determined using a bicinchoninic acid (BCA) assay [39].

### DNA extraction and qPCR

Dissected tissues were placed immediately into 2-ml bead-beating tubes containing 0.2 g of 0.1-mm silica beads and 300 μl of 1X TE buffer. Samples were bead beaten for a total of 2 min at 30-s intervals. To each sample, 100 μl lysis buffer (20 mM Tris-HCl, 2 mM EDTA, 5% Triton X-100, 80 mg/ml lysozyme, pH 8.0) was added and the samples were incubated at 37 °C for 30 min. DNA was then purified using a Thermo Fisher Scientific GeneJet Genomic DNA Purification Kit according to the manufacturer’s instructions for gram-positive bacteria.

We quantified total bacterial abundance for each of the four tissue types with a real-time PCR (qPCR) assay of 16S rRNA gene copies [40]. This assay provides significantly broader coverage than previously reported universal bacterial quantification assays. A standard curve was generated using a 10-fold serial dilution series of a plasmid standard containing a full-length *Escherichia coli* 16S rRNA gene. A 466 bp fragment in the V3–V4 region of the bacterial rRNA gene was amplified from total DNA using universal primer pair (5′-CCTACGGGDGGCWGCA-3′ and 5′-GGACTACHVGGGTMTCTAATC-3′). Quantitative PCRs were carried out on a BioRad CFX96 thermocycler in 12 μl reactions containing 9 μl of iTaq Universal SYBR Green Supermix (BioRad), 0.5 μl forward primer, 0.5 μl reverse primer, and 2 μl of DNA template. The cycling conditions were 95 °C for 3 min followed by 40 cycles of 95 °C for 10 s and 60 °C for 60 s. The assay was validated for use on honey bee-associated bacteria by confirming amplification against individual plasmid templates harboring full-length 16S genes corresponding to major gut phylotypes. The qPCR results were expressed as the total number of 16S rRNA gene copies per DNA extraction (200 μl volume elution).

### 16S rRNA gene sequencing

The V6–V8 variable region of the 16S rRNA gene was amplified using PCR primers 799F (acCMGGATTAGATACCCKG + barcode) and bac1193R (CRTCCMCACCTTCCTC). Amplification was performed using the HotStarTaq Plus Master Mix Kit (Qiagen, USA) under the following conditions: 94 °C for 3 min, followed by 28 cycles of 94 °C for 30 s, 53 °C for 40 s, and 72 °C for 1 min, with a final elongation step at 72 °C for 5 min. PCR products were confirmed using a 2% agarose gel. PCR products were used to prepare DNA libraries following Illumina TruSeq DNA library preparation protocol. Sequencing was performed on a MiSeq at the University of Arizona Genetics Core.

### 16S rRNA gene community analysis

16S rRNA gene sequences were processed using MOTHUR v.1.35.0 [41]. Forward and reverse reads were joined using the make.contigs command. After the reads were joined, the first and last five nucleotides were removed using the SED command in UNIX. Using the screen.seqs command, sequences were screened to remove ambiguous bases. Unique sequences were generated using the unique.seqs command. A count file containing group information was generated using the count.seqs command. Sequences were aligned to Silva SSUREF database (v102) using the align.seqs command. Sequences not overlapping at the same alignment coordinates, and columns not containing data, were removed using the filter.seqs command. Sequences were preclustered using the pre.culster command. Chimeras were removed using UCHIME [42], and any sequences that were not of known bacterial origin were removed using the remove.seqs command. All remaining sequences were classified using the classify.seqs command. All unique sequences with one or two members (single/doubletons) were removed using the AWK command in UNIX. A distance matrix was constructed for the aligned sequences using the dist.seqs command. Sequences were classified at the unique level with the Ribosomal Database Project (RDP) Naive Bayesian Classifier [43] using a manually constructed training set containing sequences sourced from the greengenes 16S rRNA database (version gg_13_5_99 accessed May 2013), the RDP version 9 training set, and all full-length honeybee-associated gut microbiota on NCBI (accessed July 2013). Operational taxonomic units (OTUs) were generated using the cluster command. Representative sequences for each OTU were generated using the get.oturep command. To further confirm taxonomy, resulting representative sequences were subject to a BLAST query using the NCBI nucleotide database. Diversity indices were generated using the rarefaction.single and summary.single (alpha diversity) commands.

### Statistical analysis

To examine the effect of community size, we multiplied the proportional abundance of OTUs by group or species-specific 16S rRNA gene copy number and total bacterial 16S rRNA gene copies determined with qPCR for each individual queen and niche. All core bacterial genomes contain four 16S rRNA gene copies except *L. kunkeei* (5), *Bifidobacterium asteroides* (2), and *P. apium* (1). Acetobacteraceae Alpha 2.1 (copy number unknown) was designated a value of one, consistent with the copy number of its closest relative, *P. apium* [44]. In this case, qPCR-normalized abundance is extrapolated from relative abundance of amplicons, so remains compositional. OTUs at low abundance were summed (Σ OTUs 10–500), corrected for community size and mean 16S rRNA gene copy number (4.2) [45], and used to assess the change in collective low-abundance OTUs with chronological age and biological age. As an exploration of “other” or sporadic low abundance bacteria, we present analyses with and without low abundance OTUs.

We used both parametric and non-parametric tests to analyze different properties of our data. To allow the use of parametric multivariate analyses [46], we converted the qPCR-normalized bacterial abundances to ratios among all OTUs [47] using the software CoDaPack’s centered log ratio (CLR) transformation [48]. These transformations reflect the ratio abundance of all taxa in the data set. Nearly all of these transformed data sets were normally distributed [47]. A few samples deviated slightly from normal following transformation. Because our sample size is large (*n* = 63 for each IV), these tests are robust to slight deviations from normality. As an additional measure, we used Pillai’s Trace test statistic, also robust to violations of multivariate normality and homogeneity of covariance. The MANOVA and multivariate analysis of covariance (MANCOVA) were performed on CLR-transformed data with OTUs 1–9 as dependent variables. The MANOVA examined age and source as independent variables, and post-hoc pairwise analyses were conducted using Tukey HSD. The MANCOVA explored biological age (carbonyl accumulation) as a covariate, while partitioning variation associated with chronological age and source. We compared qPCR-normalized abundance of each bacterial taxon by age without reference to source variation using the Wilcoxon rank sum test followed by Bonferroni, Benjamini-Hochberg, and FDR corrections to account for multiple comparisons.

We examined the relationship between biological aging (carbonyl accumulation) and the microbiota in various ways: (1) we performed distance-based linear model (DistLM) on the top 37 OTUs to test whether the microbiota from each of four distinct tissues is significantly associated with carbonyl accumulation in queens, (2) we examined carbonyl accumulation as a covariate in three separate MANCOVA models (above), (3) we calculated independent Pearson’s correlations between species-specific CLR scores and log transformed carbonyl data, and (4) we performed principle component analysis (PCA) on CLR scores from OTUs 1–9, plotting the relationship of bacterial community composition and age-associated succession relative to carbonyl accumulation by niche. As a confirmation of the clustering and separation produced by PCA, we performed ANOSIM on the raw amplicon reads from the top 37 OTUs to test for microbiota similarity by age and source. For the extended queen data set, we calculated correlations among the top 200 OTUs using sparse correlations for compositional data algorithm (SparCC; [49]) as implemented in mothur [41]. SparCC is robust for compositional data sets with a low effective number of species [50]. Analyses were conducted in JMP_ v11 (JMP_ 1989–2007) and/or SAS_ v9.4 [51].

To compare the microbiota of aging queens with those of aging workers, we analyze worker age data from a recently published manuscript [19]. As one of three studies sequencing both nurses and foragers with the same primer sets and PCR parameters, the Kwong et al. study is the most robust and provides whole gut microbiota values based on 16S rRNA gene sequences from differently aged workers of *Apis mellifera*, *n =* 84 workers, 19 foragers (old) and 65 in-hive bees (younger). From this data set, we designated eight core gut bacteria representing 95% of OTU abundance based on known samples of whole gut communities in the literature. The remaining 5% OTU abundance from [19] was comprised primarily (83%) of Proteobacteria, occurred with sporadic abundance and prevalence, and was combined as an “other” category to represent low-abundance bacteria. As stated above for queens, we CLR-transformed relative abundance measures of workers and performed a one-way MANOVA on age to compare old vs. young gut microbiota, calculating post hoc differences between specific bacterial groups. To visually compare the queen and worker results, we transform our tissue-specific queen data to reflect relative abundance values predicted for the whole gut. Tissue-specific bacterial cell counts were used to normalize the relative occurrence of bacterial species by queen tissue then additively produce a single value that represents the expected result of sequencing whole queen guts. These whole gut values highlight hypothetical differences in abundance and prevalence between workers and queens.

## Results

### 16S rRNA gene sequencing and qPCR

Next-generation sequencing returned 7.2 million quality trimmed reads (400 bp assembled) across the 252 libraries (Additional file 1: Table S1). Only four mouthpart libraries were excluded from the analysis, and read coverage in the remaining 248 libraries was sufficient for all downstream characterization and statistics (Additional file 2: Table S2). The queen rectum was represented by 2.4 million reads averaging 38 K per library, the ileum by 1.9 million reads averaging 30 K per library, the midgut by 1.6 million reads averaging 25 K per library, and the mouth by 1.3 million reads averaging 21 K per library. Like the gut microbiota of workers, that of queens is taxonomically simple and represented by only 7–12 conspicuous OTU’s dependent on niche. The remaining OTUs were highly sparse and typically at less than 10% prevalence (Additional file 1: Table S1). The nine most common 97% OTUs accounted for 98.8% of all reads across the combined niches. Given the low effective number of OTUs, unique OTUs were manually assessed to verify 97% species clusters. Subtracting the rare biosphere (1.2%), these nine OTUs are the focus of most analyses and are what we present in figures. Summed across the four niches, the nine most abundant OTUs according to raw amplicon read totals were *Lactobacillus* firm5 species cluster (51.3%), *Parasaccharibacter apium* (27.1%), *Lactobacillus kunkeei* (7.6%), L*actobacillus* firm4 species cluster (6.8%), *Acetobacteraceae* Alpha 2.1 species cluster (2.0%), *Bifidobacterium asteroides* (1.5%), *Snodgrassella alvi* (1.8%), *Gilliamella apicola* (0.3%), and *Delftia* spp. (0.2%). In honey bees, *Delftia* is genus of Burkholderiales not recognized in workers.

Similar to the abundance pattern in worker guts, the queen rectum harbors an average of 121.2 M 16S rRNA gene copies, a magnitude more than the ileum (17.9 M) or midgut (14.2 M). The mouth (1.4 M) contains the least bacteria. Total amplicon reads returned for the mouth, midgut, and ileum were significantly correlated with bacterial abundance as determined by qPCR (Additional file 2: Table S2). OTU dominance in the queen increased with community size in the mouth, midgut, and ileum (Additional file 2: Table S2). Extrapolating qPCR results to estimate community size, *P. apium*, and *L. kunkeei* decrease in abundance approaching the rectum, while L. firm5, L. firm4, *B. asteroides*, and Alpha 2.1 increase (Additional file 3: Table S3). *S. alvi* and *G. apicola* occur sporadically at low (< 1%) average relative abundance throughout all queen niches.

### MANOVA of queen microbiota by chronological age and source

**Fig. 1:**
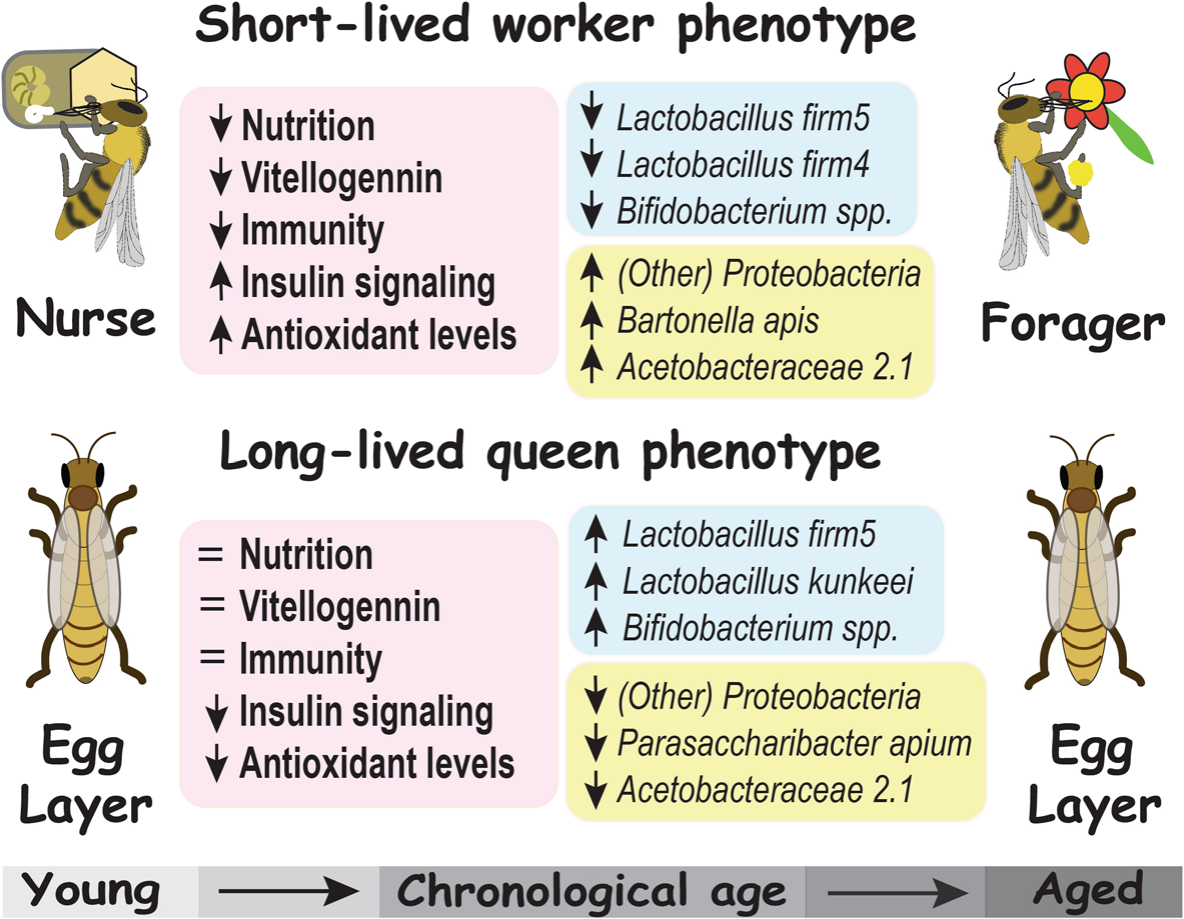
Age-associated bacterial succession of distinct longevity phenotypes. Honey bee host differences (pink panels) reflect aging physiology. In the context of life history theory, workers are literally the “disposable soma,” while queens represent reproduction [10]. Vertical arrows indicate the direction of change with increasing age. *Lactobacillus* and *Bifidobacterium* are listed in the blue panels and Proteobacteria in the yellow panels. All listed bacterial groups differ significantly in ratio abundance. The microbiota of the short-lived worker phenotype represents a meta-analysis of *Apis mellifera* gut libraries from Kwong et al. [19]. Queens were analyzed in the present study (see Table 1)

**Fig. 2:**
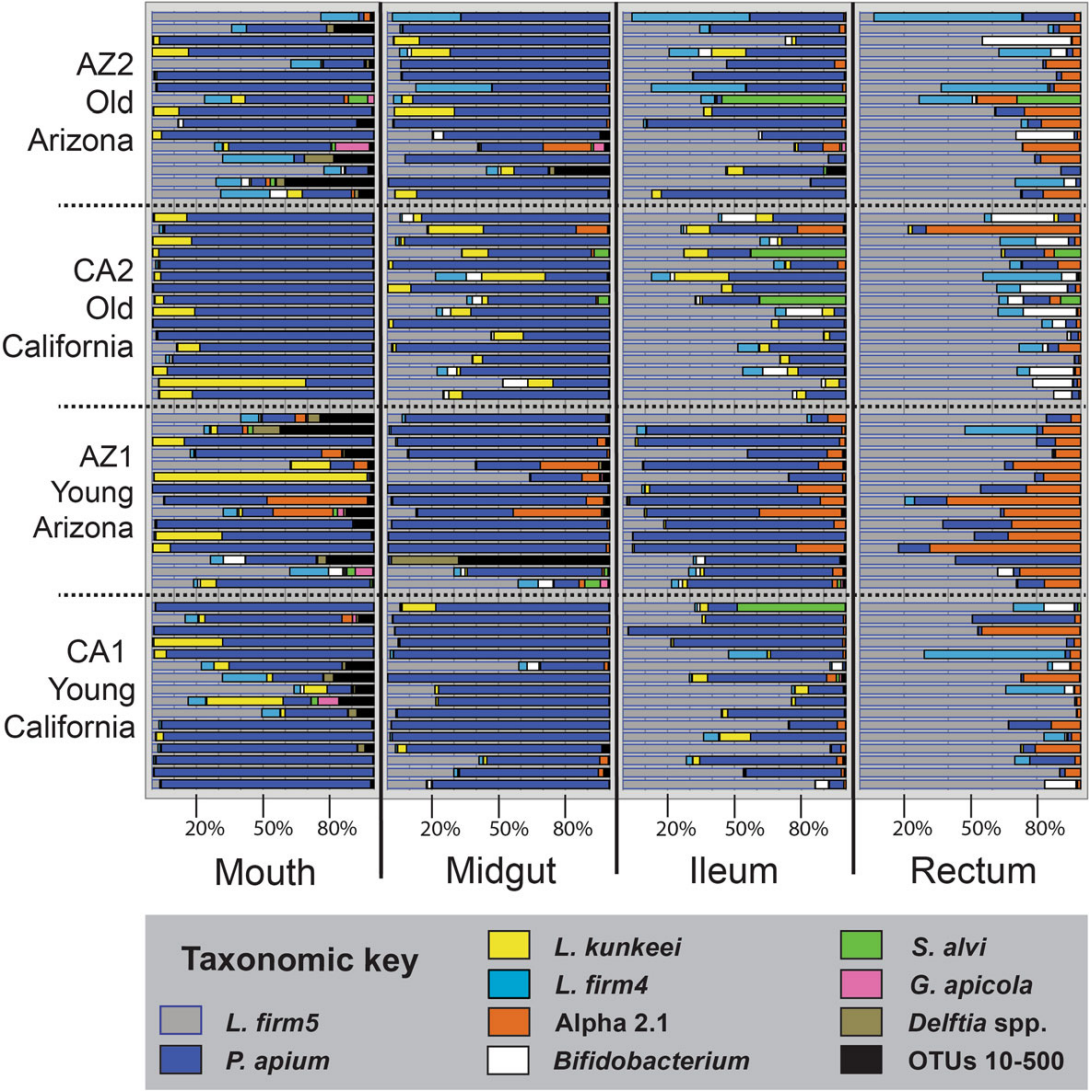
The honey bee queen microbiota by tissue. Color-coded bars represent relative abundance corrected by species-specific 16S rRNA gene copy number (see Additional file 3: Table S3 for normalized abundance). The 4 × 4 panel displays the top nine most abundant OTUs by niche, age, and source. Black represents the summation of OTUs 10–500. Old queens in the upper two rows are 16–18 months of age and young queens in the bottom two rows are aged 4.5–5.7 months (Fig. 4)

The two-way MANOVA performed for each of the four queen niches revealed significant variation due to chronological age, source, and interaction (Table 1, Additional file 4: Table S4). In the mouth, *P. apium* and L. firm5 increased with age, while Alpha 2.1 and *Delftia* were more abundant in young queens. The midgut and ileum aged similarly; in both niches, *B. asteroides* and *L. kunkeei* were more abundant in old queens while Alpha 2.1, *Delftia*, and “other” all decreased with age (Fig. 1). Most abundant in the ileum relative to other queen tissues, *S. alvi* bloomed in 4 of 63 individuals and increased with age, while *P. apium* and *G. apicola* decreased (Additional file 3: Table S3). In the rectum, where L. firm5 represents the majority of total gut bacteria, *B. asteroides* abundance increased with age while L. firm5 and both core *Acetobacteraceae* (*P. apium* and Alpha 2.1) decreased (Fig. 2). Wilcoxon rank sum tests revealed significant differences by chronological age, many of which agree with age-specific differences detected in the two-way MANOVA (Table 1, Additional file 4: Table S4). Wilcoxon tests provide context comparing chronological age without regard to environmental source (Additional file 5: Table S5).

**Table 1:** Queen microbiota: results by niche, chronological age, source and biological age

Category, group, or OTU^a^	Abundance^b^ low or high	Percent change w/ age^c^	Wilcoxon rank sum test^d^	MANOVA^e^	
				F value	Pr ≥ F
Mouth					
*Lactobacillus* firm5	H	+ 59	0.05	–	*ns*
*Lactobacillus firm4	L	+ 195	0.05	–	*ns*
Parasaccharibacter apium**	H	+ 341	0.05	–	*ns*
*Acetobacteraceae* Alpha2.1	H	− 97	*ns*	7.8	0.007
*Snodgrassella alvi*	L	+ 121	0.05	5.6	0.02
*Delftia* (*Burkholderiales*)	L	− 53	0.0009	12.4	0.0008
Midgut					
Bifidobacterium asteroides**	L	+ 242	0.03	14.1	0.0004
Lactobacillus kunkeei**	H	+ 336	0.05	5.2	0.03
*Acetobacteraceae Alpha2.1	L	− 79	0.03	**–**	*ns*
*Delftia (*Burkholderiales*)**	L	− 93	0.0009	26.2	< 0.0001
Ileum					
Bifidobacterium asteroides**	H	+ 164	*ns*	5.9	0.02
*Lactobacillus* firm5**	H	+ 9	*ns*	–	*ns*
*Lactobacillus kunkeei	H	+ 248	*ns*	6.4	0.01
*Acetobacteraceae* Alpha2.1	H	− 90	0.005	7.1	0.01
*Parasaccharibacter apium	H	− 50	0.02	–	*ns*
*Snodgrassella alvi	H	+ 279	*ns*	11.42	0.001
*Gilliamella apicola**	L	− 69	0.05	–	*ns*
*Delftia* (*Burkholderiales*)**	L	− 94	0.0009	36.2	< 0.0001
Rectum					
Bifidobacterium asteroides**	H	+ 212	0.005	15.5	0.0002
*Lactobacillus* firm5	H	− 33	0.03	9.3	0.004
*Acetobacteraceae* Alpha2.1	H	− 76	0.009	–	*ns*
*Parasaccharibacter apium**	H	− 78	0.04	11.7	0.001
*Snodgrassella alvi	L	+ 984	0.006	11.4	0.001
*Delftia (*Burkholderiales*)**	L	− 92	0.0009	35.7	< 0.0001

^a^Dependent variables are OTUs 1–9 normalized by community size (qPCR) and 16S rRNA gene copy number

^b^Low abundance (L) = < 1% mean bacterial cell number by niche (Additional file 2: Table S2)

^c^Average percent change in bacterial cell number with age. We note that cell number loss cannot exceed 100%

^d^Wilcoxon rank sum test with FDR correction comparing normalized bacterial abundance by chronological age only (Additional file 5: Table S5)

^e^Independent variables are queen chronological age and source (df = 3, 59). Reports only F values for chronological age effects examining the top nine most abundant OTUs

*Significant interaction effect of chronological age and source detected by the two-way MANOVA (Additional file 4: Table S4)

**Significant MANCOVA result and Pearson correlation of bacterial abundance with biological age (Additional file 8: Table S8 and Additional file 9: Table S9)

*ns* not significant

### MANOVA of worker microbiota by age

**Fig. 3:**
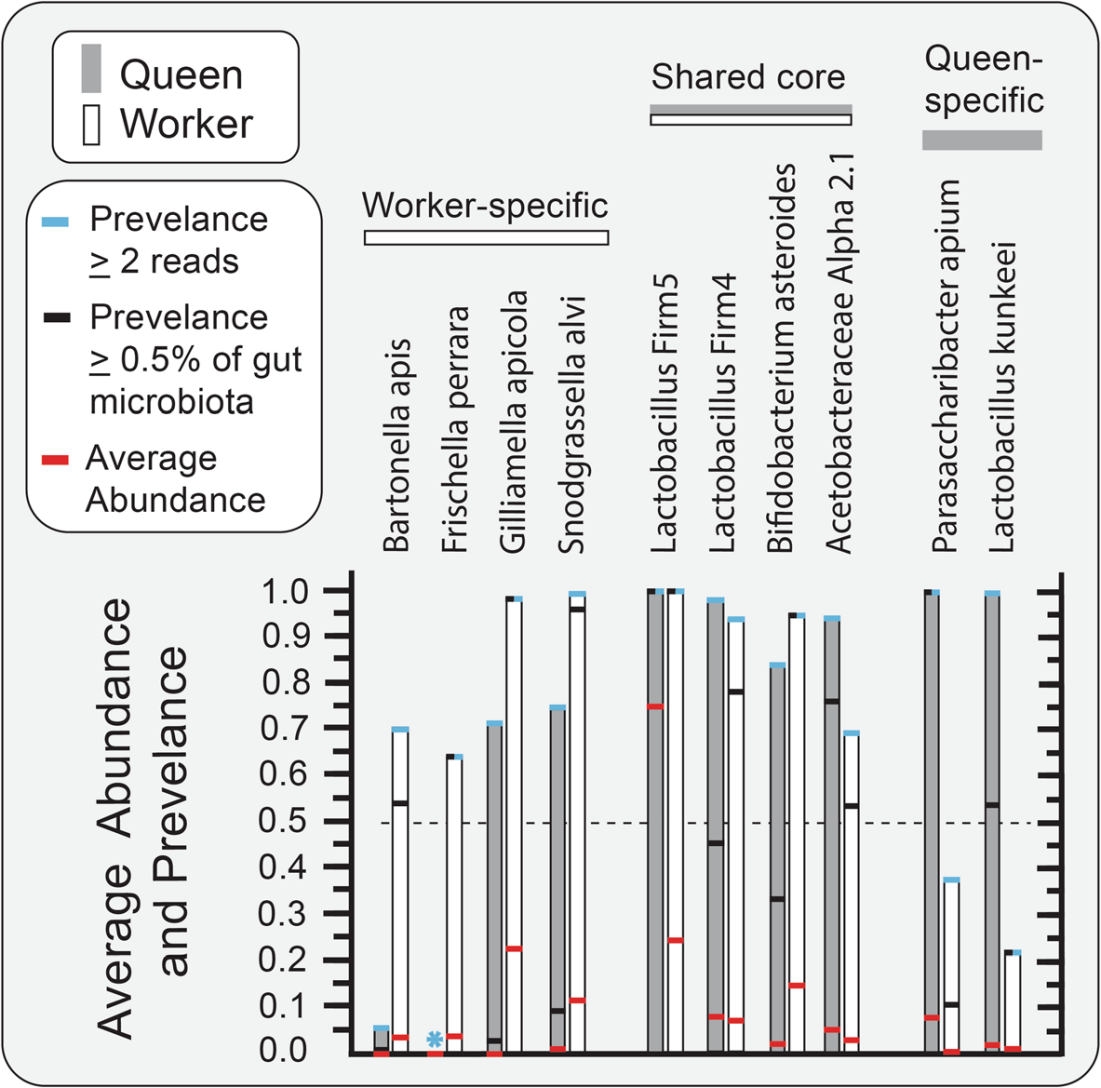
Average abundance and prevalence of gut bacteria in queens (*n* = 63) and workers (*n* = 83). Workers are whole gut samples from Kwong et al. [19]. Queen data was normalized by tissue-specific community size to reflect relative abundance values expected from sampling whole guts. The red bars represent average abundance, black bars are prevalence defined at ≥ 0.5% relative abundance, and the bar apex is prevalence defined as two or more reads per gut library. We did not detect F. perrara* in any of the four sampled queen alimentary tract niches

Analyzing data published in Kwong et al. (2017), we investigated age-associated changes in the gut microbiota according to the behavioral role of the worker. The one-way MANOVA of microbiota by worker age (task) revealed major proportional shifts among core gut bacteria (Fig. 1). *Bifidobacterium*, L. firm5 and L. firm4 decreased significantly with age while “other” bacteria, Acetobacteraceae Alpha 2.1, and *Bartonella apis* increased (Additional file 6: Table S6). Of note, *S. alvi* decreased in relative abundance with age but was borderline insignificant (*p* = 0.06). With reference to these results, and other worker data sets in the literature [19], we use a cutoff value of 50% prevalence at 0.5% average relative abundance to define bacterial taxa specific to each longevity phenotype. Applying this value, we identify four worker-specific gut groups, all Proteobacteria, two queen-specific groups, and four groups shared by longevity phenotypes (Fig. 3). In general, the four bacterial groups with fidelity for the rectum are shared by longevity phenotypes while the ileum-specific groups differ by longevity phenotype.

### Biological age, source, and the queen microbiota

**Fig. 4:**
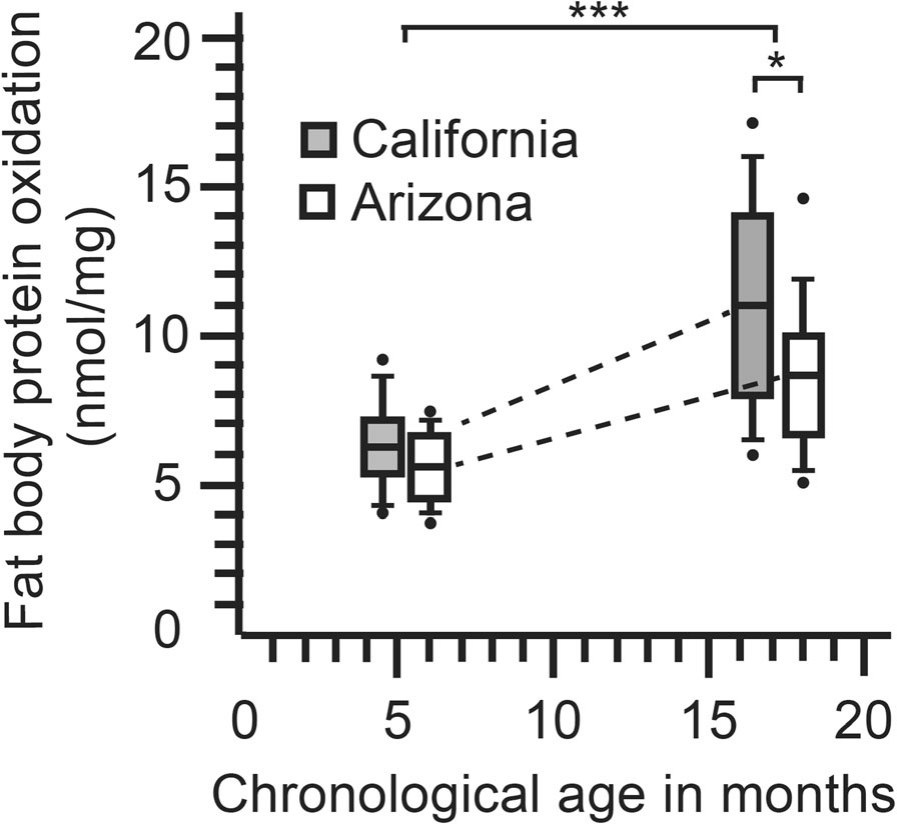
Carbonyl accumulation (protein oxidation) in queen fat body differs by chronological age (F_3, 59_ = 48.3; *P* < 0.0001***) and source (*t* = 2.2; df = 30, *P* = 0.03*)

We measured carbonyl accumulation in the queen fat body as a proxy for queen biological age. While some variation in carbonyl accumulation is due to genetics and environment, difficult to excrete waste products accumulate in a clock-like fashion with age. We found that chronological age was strongly associated with carbonyl content in the fat body of the queen (Fig. 4). Carbonyl accumulation differed by both chronological age and source (Additional file 7: Table S7). Examining all pairwise combinations, only first year queens (CA1 and AZ1) did not differ in average carbonyl accumulation. In both sets of young and old queens, chronological age did not agree with biological age. In both age classes, queens from the Imperial Valley of California (source CA) were chronologically younger, but biologically older with greater carbonyl accumulation (Fig. 4). The finding that biological age differed significantly from chronological age suggests that “source” (environmental exposure and genetic background) is a major contributor to biological age and is a confounding factor for the analysis of chronological age. For this reason, we emphasize the statistical tests and interpretations that control for source variation.

**Fig. 5:**
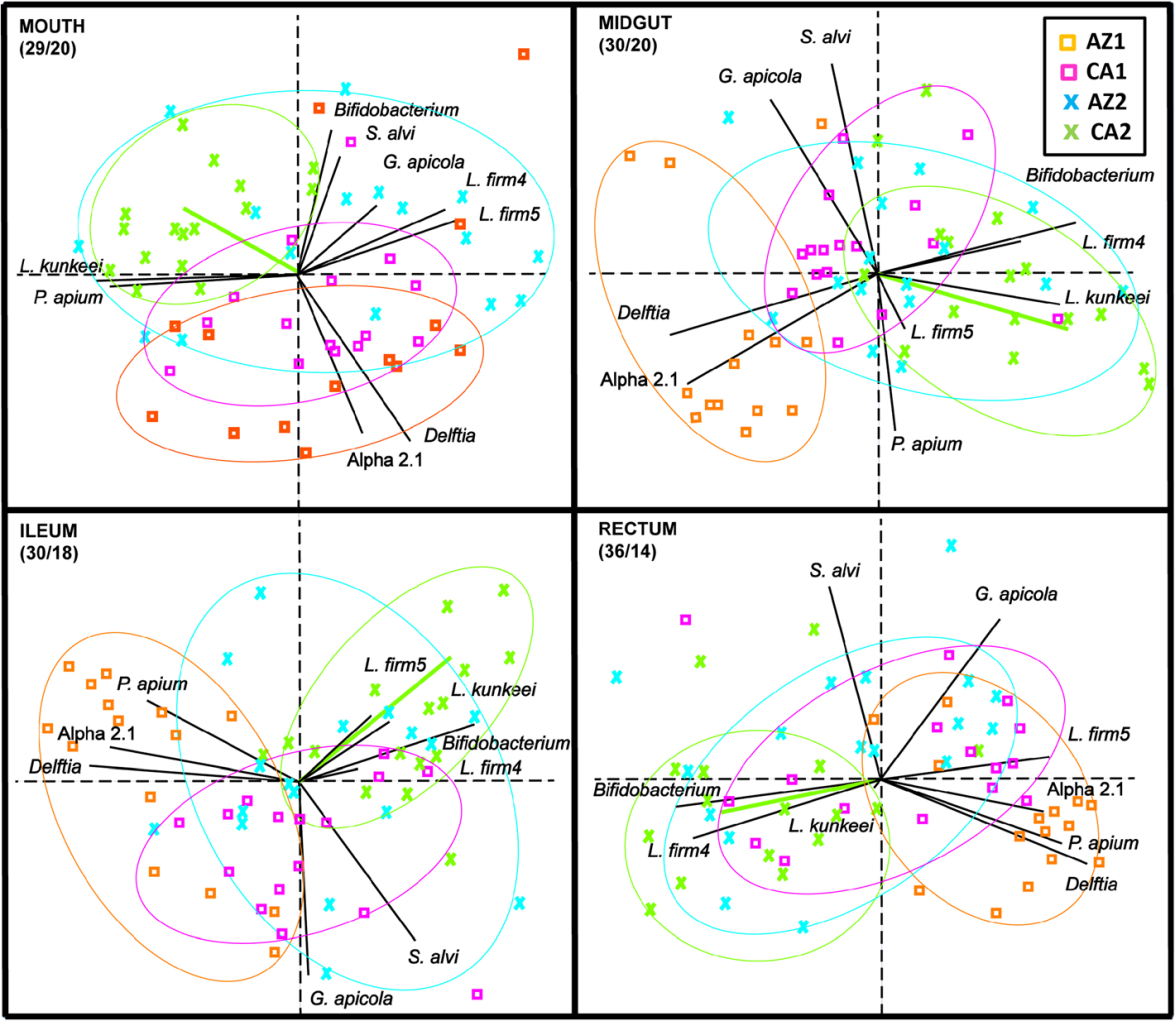
Principle components analysis by niche based on the top nine most abundant OTUs and carbonyl accumulation. The colored symbols illustrate differences among the chronological sample cohorts; pink and orange are young and blue and green are old. The green vector illustrates carbonyl accumulation relative to community structure, shows strong affinity with increased *Lactobacillus* and *B. asteroides* in the gut, and is largely allied with the biologically oldest queen cohort (CA2). Orange symbols are biologically the youngest and consistently allied with *P. apium* in the hindgut and Acetobacteraceae Alpha 2.1 and *Delftia* throughout the system. Biplot constructed with normalized bacterial cell abundance data, transformed to centered log ratios (CLR) that represent the change in taxon abundance (covariance) relative to all other taxa in the data set. The species vectors are proportional to the standard deviation of the ratio of each taxon to all other taxa. In general, clustered groups of points contain similar groupings of taxa with similar ratio abundances, and longer OTU vectors result from greater variation in CLR scores. The parentheses below each niche label contain the percent variation explained by the first and second principle component respectively (Additional file 10: Table S10)

To further explore the relationship of biological age and source variation with queen microbiota, we ran a set of related analyses that partition variation by different strategies. DistLM revealed a significant association between microbiota composition and carbonyl accumulation in each of the four tested communities (Mouth, pseudo-F_61_ = 4.1: *P* = 0.01; midgut, pseudo-F_61_ = 3.7: *P* = 0.006; ileum, pseudo-F_61_ = 4.1: *P* = 0.004; rectum, pseudo-F_61_ = 3.9: *P* = 0.005). Although all four communities were significantly associated with carbonyl accumulation, little variation was explained by the collective community (mean R-sq = 0.06) due to opposing species variation within communities. The separation of background (source), chronological age, and carbonyl accumulation via MANCOVA analyses detailed OTU-specific changes in the microbiota (Additional file 8: Table S8). Pearson’s correlations examining species-specific CLR log-transformed OTU abundance and log-transformed carbonyl values agree with the main MANCOVA results examining source as the dependent variable with carbonyl accumulation as the covariate without reference to chronological time (Additional file 9: Table S9). Most notably, throughout the gut, *B. asteroides* was correlated significantly with the accumulation of carbonyl in abdominal fat body tissue (Fig. 5). Although at similar abundance in chronologically old and young queens, L. firm5 abundance was also correlated strongly and positively with carbonyl accumulation. Rare throughout the queen gut, an undescribed Burkholderiales, *Delftia* spp. showed the strongest negative relationship with carbonyl content, decreasing dramatically with age and varying by source (Table 1).

To visualize variation associated with biological age in the queen microbiota, we performed PCA analysis using centered log ratios from the top nine OTUs and associated carbonyl values from the fat body of each queen (Fig. 5, Additional file 10: Table S10). Across each niche, the first two principle components explained approximately 50% of the variation in log ratio abundance scores. Because the queen microbiota has shallow, deep, and noisy structure, the third and fourth principle components for each niche explained an average of 14 and 8%, respectively (Additional file 10: Table S10). Although only 50% of the variation is presented in the two dimensional PCAs, a strong and consistent separation of two queen cohorts is realized in every niche, young Arizona (AZ1) and old California (CA2). In each niche, the carbonyl vector indicates CA2 as the oldest, and AZ1 as the youngest cohort, consistent with determinations of biological age. Also consistent with results for biological age, ANOSIM results support the PCA clustering and separation (Additional file 11: Table S11).

We examined microbiota correlations using SparCC, an approach that incorporates the structure of the data matrix to identify potential species interactions and generates null expectations based on permutation of OTU columns in the transformed data matrix. Based on SparCC, the mouth and midgut reveal a number of significant positive relationships between core bacteria within niche (Additional file 12: Table S12). We note that SparCC results are unreliable when OTU sparsity exceeds 70% zero values but robust to communities with a low effective number of species [49] (see Table 1). The ileum reveals a marked decrease in positive relationships and the first occurrence of significant negative relationships. As the relevant dynamic, the two major *Acetobacteraceae* (Alpha 2.1 and *P. apium*) associate positively in the ileum, but both associate negatively with L. firm5 and *Bifidobacterium*. The strongest negative correlation occurs between L. firm5 and *P. apium*, the two most abundant ileum species (Additional file 12: Table S12). With more detailed investigation, age-specific Pearson’s correlations on log-transformed normalized abundance shows that as queens age, the relationship of L. firm5*/P. apium* cell number shifts from mildly negative (Pearson’s *r* = − 0.27, *p* < 0.07) to strongly positive (Pearson’s *r* = 0.49, *p* < 0.005), concurrent with the loss of *Acetobacteraceae* (Alpha 2.1 and *P. apium*).

## Discussion

Our results support the hypothesis that gut microbiotas of queen and worker longevity phenotypes differ in structure and membership and show age-associated changes concurrent with host physiology, diet, and behavior (Fig. 1). Because long- and short-lived phenotypes are produced from the same genotype, microbiota establishment and age-associated changes likely reflect host regulatory anatomy and environmental exposure, primarily diet. Long-lived (queen) phenotypes are fed royal jelly throughout their lives to replenish internal levels of vitellogenin. In their youth, short-lived (worker) phenotypes consume pollen to produce a discrete pulse of vitellogenin that fuels royal jelly synthesis in their head glands. In old age, workers forage for pollen and nectar consuming honey to support flight metabolism. This fundamental difference in diet and behavioral task reflects a suite of age-associated host gene expression, highlighted by differences in vitellogenin production, immunity, insulin signaling, and antioxidant levels [9, 14, 16, 25, 52, 53]. These core changes in host physiology are consistent with the distinct microbiota compositions and age-based succession of honey bee longevity phenotypes (Fig. 1). In general, aging worker guts show decreased *Lactobacillus* and *Bifidobacterium* and increased Proteobacteria adding to the list of insect and mammal systems where this pattern has been documented [30]. In stark contrast, the gut microbiota of aging queens is depleted of core and other Proteobacteria and accumulates core hindgut bacteria typically considered probiotic like *Lactobacillus* and *Bifidobacterium*.

We tracked chronological queen age, but also quantified carbonyl accumulation in the queen fat body as a marker of biological aging (Fig. 4). We hypothesize that the portion of microbiota variation best explained by biological age may be associated with environmental variables including diet quality. Vitellogenin is the major fat body lipoprotein and plays an essential role in oxidative stress resistance and honey bee lifespan [14, 18]. The rate of vitellogenin synthesis in laying queens is 20 times that of workers, and much of the oxidative damage in queens is absorbed by vitellogenin [14, 18]. Changes in diet quality or other environmental variables that influence colony homeostasis (i.e., overheating and intensive water collection) may affect the production and quality of royal jelly produced by workers and fed to the queen. In turn, the royal jelly diet provides defense against oxidative damage in the queen gut and fuels the production of vitellogenin throughout the queen hemolymph. Similarly, workers deprived of pollen are extremely low in vitellogenin and do not develop a normal microbiota suggesting that pollen serves as a prebiotic in the worker gut [24] and/or that the regulatory anatomy associated with the conversion of ingested pollen to vitellogenin is intimately linked to the development of a normal gut microbiota [25].

### Longevity phenotypes differ in core membership

Six of the gut bacterial groups (or species) show strong specificity by longevity phenotype (Fig. 3). The four Proteobacteria we classified as worker-specific show distinct patterns of rarity in queen guts. In an evolutionary context, the two most recent additions to the worker gut microbiota are *B. apis* and *Frischella perrara* [19]. Perhaps a result of this novelty, these bacteria exhibit a relatively narrow niche breadth. *F. perrara* is specific to the worker pylorus and results in host melanization response, while *B. apis* appears in the hindguts of older foragers [28, 54–56]. In queens, *B. apis* was extremely rare and *F. perrara* not detected, not even on the mouth, suggesting that these particular Proteobacteria are not tolerated by the queen or excluded via some mechanism perhaps the antimicrobial mechanisms of royal jelly. In contrast, worker-specific *G. apicola* and *S. alvi* are tolerated at low levels in queen guts, and *S. alvi* showed sporadic abundance in 4 of 63 seemingly healthy queen ileums (Fig. 2). In workers, this species pair is omnipresent, accounts for 20–60% of the ileum microbiota, and represents a core syntrophic relationship critical to gut oxygen balance [25, 26, 57, 58]. *G. apicola* produces acetate under low oxygen, and *S. alvi* is an obligate aerobe that consumes acetate to fuel its oxygen metabolism [25]. Although at low abundance in queens, this species pair is highly correlated throughout all sampled queen niches occurring with ≤ 10% prevalence defined at 0.5% average abundance (Fig. 3). Queen-specific bacterial species are *Parasaccharibacter apium* and *Lactobacillus kunkeei*, both showing strong fidelity for queen mouth, midgut, and ileum*.* These two species may be associated with the royal jelly diet of the queen, but occur with sporadic abundance in worker ileums under conditions of putative dysbiosis and oxidative stress [12, 23, 29, 34].

The four bacterial groups shared by queens and workers differ in abundance and prevalence showing strong niche fidelity (Fig. 3). Of these four, only *Lactobacillus* firm5 is core to both the ileum and rectum of both longevity phenotypes. Considering whole guts independent of age, queens and workers average 75 and 24% relative abundance of *Lactobacillus* firm5, respectively. Three of the four shared core groups (L. firm5, L. firm4, and *B. asteroides*) populate 100% of workers by 3 days of age [22]. Of these three, *B. asteroides* is significantly more abundant and prevalent in workers than queens (particularly young workers), perhaps associated with pollen consumption [24, 59]. However, *B. asteroides* increases significantly in the hindguts of aging queens independent of pollen consumption. The fourth shared group, *Acetobacteraceae* Alpha 2.1, is abundant in young queens but not typically detected in young workers. It decreases with queen age but becomes prevalent and abundant in older workers [19, 31, 60–64].

### The queen microbiota improves with age

Our results are consistent with the body of work detailing biological aging and oxidative stress in queens, workers, and social insects in general [8–10, 65]. Results from the queen carbonyl assay demonstrate that queens accrue oxidative damage with age (Fig. 4) and that chronological age can differ significantly from biological age possibly due to environmental differences including climate, nutrition, toxins, and other landscape variables. Despite similar signs of biological aging in both queens and workers, the aging gut microbiota of older queens seems to reflect a refined structure with greater efficiency. It is unlikely that queens ever develop a senescence physiology and associated microbiota as seen in workers. Under natural conditions, queens accrue biological damage associated with aging, but are not allowed to grow “old” because fecundity is critical to colony survival, and workers routinely replace substandard queens [66]. With increased oxidative damage, gram-positive bacteria decrease in workers [19] but increase in queens (Fig. 1). Of note, core *Lactobacillus* and *B. asteroides* in queens show greater correspondence with biological than chronological age suggesting that these species may track and/or signal host physiology (Additional file 8: Table S8). Consistent with decreased antioxidant expression and less generation of reactive oxygen species in queens [9, 65], the bacteria that increase with queen age do not rely on oxygen, but generate continuous fermentative metabolism in the queen hindgut (Fig. 2). In turn, these fermentation products (i.e., butyrate) are considered fundamental to host physiology and homeostasis [25, 59].

Results from conventionalized bee experiments suggest that butyrate produced by the honey bee worker hindgut microbiota plays a key role in host metabolism [25, 59]. In human colons, positive butyrogenic effects are considered a result of cross feeding by butyrate-producing Firmicutes and *Bifidobacterium* [67]. Feeding worker honey bees’ relevant amounts of sodium butyrate results in gene expression considered beneficial to general health, broadly affecting immunity and detoxification [68]. We found that bacterial communities implicated in butyrate production were diminished in aging workers but seemingly enhanced in aging queens. Better explained by biological than chronological age (Fig. 5), *B. asteroides* increased significantly with age in the queen midgut, ileum, and rectum (Additional file 8: Table S8 and Additional file 9: Table S9). Moreover, *L. kunkeei* increases in the midgut and ileum, while community changes in the ileum favor the persistence of *Lactobacillus* firm5 (Table 1), and suggests a more efficient relationship that emerges with queen age. *Lactobacillus* firm5 is the most plentiful bacteria in the queen hindgut, and combined with increased *B. asteroides*, may add to the butyrogenic effect in queens concurrent with increased biological age. *B. asteroides* itself was recently identified as a major bacterium associated with host-derived signaling molecules in worker honey bees [59]. Interestingly, *B. asteroides* abundance in both queens and workers is often low and/or highly variable so may be affected by diet or strain variability [24].

We compared the gut microbiota of young in-hive bees to older foragers within and among studies. Foraging is the last functional role workers serve before death. But as a behavioral cohort, both in-hive bees and foragers can range greatly in chronological age and environmental exposure [69]. Also, comparing across next-generation sequencing studies can be misleading due to differences in methodology like primer choice or analysis pipeline [70]. Despite these and other sources of potential error, we found that the worker gut microbiota ages in a highly predictable fashion, becoming depleted of core hindgut *Lactobacillus* firm4, firm5, and *B. asteroides* (Fig. 1). Of seven available studies sequencing the 16S gene from forager guts, three used the same primers and methods to sequence both foragers and nurses [19, 31, 63], and we used these studies as a point of reference for examining worker aging. We analyzed the largest and most variable of these three data sets [19] defining six significant differences in microbiota between young and old workers (Fig. 1). The collective results from six of seven studies are largely in agreement and suggest that age-associated shifts in worker microbiota are strongly predictable despite study particulars including the use of different primer sets [19, 31, 60–64]. The changes we report in Fig. 1 [19] represent a functional change from a fermentative to proteolytic hindgut environment, involving significant shifts in core bacterial structure. *Acetobacteraceae* Alpha 2.1 increases in all seven studies, *B. apis*, and “other” bacteria in 6 of 7. One to three major core hindgut bacteria are depleted significantly in six of seven studies, while studies were more variable concerning shifts of *S. alvi* and *G. apicola*, the species pair that dominates the worker ileum.

### Early gut succession

Similar to workers [37], the rectum of the mature queen contains 84% of the total bacteria found in the queen gut (Additional file 2: Table S2). On average, a whole gut sample from a mature laying queen would be highly biased toward rectum species, dominated by *Lactobacillus* firm5 (Fig. 3). In contrast, whole gut samples of queens during the mating process show a dominant *Acetobacteraceae* (*P. apium* and Alpha 2.1) profile [20]. This finding is consistent with our detection of significantly more *P. apium* and Alpha 2.1 in the guts of younger queens (Fig. 2, Table 1) and suggests that the bacterial succession leading to a *Lactobacillus* firm5 dominant hindgut in queens may require many weeks, perhaps months. Given that worker gut succession occurs throughout the life of the worker [12, 22], we speculate that the early queen microbiota [20] represents a pioneer community that primes the gut environment or host immune system and/or potentially aids disease prevention during the days-long mating process that involves queen flight metabolism and mating with > 20 males. A successfully mated queen is fed massive amounts of royal jelly as she begins to lay eggs. The decrease and stabilization of cell replacement rate in early queen midguts [71] suggests a more stable gut environment emerges around 40 days of age, perhaps influencing bacterial succession.

### Queen niche breadth

In queens, the occurrence patterns and numerical dominance of *P. apium* in the mouth and midgut and *Lactobacillus* firm5 in the ileum and rectum suggests that the extended gut structure is important for host function (Fig. 3). The antimicrobial properties of royal jelly may be more manifested in the mouth and midgut. The taxonomic shift at the pylorus demarcates a steep physiological gradient in the adult bee gut. Recently characterized in workers, this change occurs just upstream of the ileum where Malpighian tubules feed host waste products back into the gut, and microoxygenic and pH gradients affect bacterial establishment and persistence [25, 29, 54]. Host excretions provide a different niche for bacterial co-evolution including an influx of nitrogenous waste compounds, a decrease in oxygen availability and lower pH [25]. While the effect of pollen consumption on host signaling has been investigated in workers [24, 25], the effect of the queen’s diet (royal jelly) on gut physiology and host signaling remains unknown. The reliable and predigested nature of the queen diet may generate very different collection of waste products, supporting hindgut bacterial strains distinct from those found in workers.

It is mostly unknown why queens can resist many worker diseases and vice-versa. Early queen death or rejection by the colony has become more common [66, 72], and defining disease states in queens will rely in part on the structure and function of native gut bacteria [12]. Although rare throughout the gut, the occurrence pattern of *Delftia* (Burkholderiales) suggests it could be detrimental. Not typically detected in workers, *Delftia* is negatively correlated with *Lactobacillus* firm5 and *B. asteroides* in the queen hindgut, shows the greatest negative correlation with carbonyl accumulation, and decreases significantly with biological age (Table 1). Congruently, *Delftia* is negatively correlated with putatively beneficial bacteria on the queen mouth and midgut (Additional file 9: Table S9 and Additional file 10: Table S10). These two niches are dominated by distinct sequovars of *P. apium*, a bacterium co-evolved to thrive on royal jelly [7]. Over 95% of the mouth/midgut bacteria classify as *P. apium and L. kunkeei*, both associated with decreased abundance of honey bee-specific disease caused by bacteria and microsporidia [73, 74]. One primary function of microbes in the queen mouth and midgut may be the exclusion of opportunistic and disease causing microbes. Mouth communities not dominated by *P. apium* are much smaller in size and contain significantly more *Delftia*, OTU diversity, and “other” bacteria (Additional file 3: Table S3) suggesting that *P. apium* dominance in the queen mouth and midgut limits the occurrence of detrimental bacteria in the hindgut. Older queens have significantly more *P. apium* on their mouths and *L. kunkeei* in their midguts that may accrue with age and/or improve queen hygiene promoting queen survival (Fig. 2). Pollen exposure and consumption may render workers more vulnerable than queens to frequent pathogen invasion. The queen and her constant diet of royal jelly may discourage novel microbial acquisition and provide a strong selective environment for the evolution of niche specialists. The constant consumption of royal jelly likely represents a form of purifying selection, perhaps even an arms race at the front end of the queen, producing fierce competition among *P. apium* strains for this constant and complete nutrient source.

### Evolution of “queen-specific” gut bacteria

*P. apium*, *L. kunkeei*, and close ancestors occur throughout solitary and social bees and may even predate the evolution of corbiculate bees [19, 75–79]. Both *P. apium* and *L. kunkeei* grow at extreme sugar concentrations and royal jelly enhances the in vitro growth of some strains [7, 33]. The evolution of host behavior to mechanically concentrate nectar sugars via evaporation was likely a key innovation producing strong selection for these two osmotolerant symbionts. Bacteria adapted to survive in concentrated nectar of solitary bee provisions were well positioned to develop greater fidelity with the host gut. The mature worker ileum is dominated by core bacteria *S. alvi* and *G. apicola* that co-occur in a biofilm with lesser amounts of *Lactobacillus* firm5 [12]. In contrast, the mature queen ileum is dominated by *Lactobacillus* firm5 that co-occurs with lesser amounts of core gut bacteria *P. apium* and *L. kunkeei* (Fig. 2). Reciprocally, worker ileum bacteria *S. alvi* and *G. apicola* are found at similarly low levels in the queen ileum and show sporadic abundance in the queen. These symmetrical occurrence patterns suggest antagonistic co-evolution of caste-specific gut bacteria, a hypothesis consistent with host age phenotype and development-specific pathogen strategies.

Over 16 *L. kunkeei* genomes have been compared, revealing core functionality and a large variety of accessory protein clusters that characterize different strains [80]. Isolated from the gut of *A. mellifera*, strains MP2 and EFB6 of *L. kunkeei*, were most related and differ from other *L. kunkeei* in possessing genes implicated in gut colonization including cell adhesion, biofilm formation, and horizontal transfer [35, 80]. These may represent strains that colonize the queen midgut and ileum. They may also colonize gut environments of workers and larva. That many of the *L. kunkeei* genomes lack gut-specific genes suggests they may lead more opportunistic life cycles within the hive and pollination environment. Similarly, the genome of *Parasaccharibacter apium* also reveals multiple functional traits for biofilm life in the insect gut, including survival in low oxygen environments and adhesion to host epithelium [44]. Like *S. alvi*, and many other acetic acid bacteria, *P. apium* can grow aerobically and assimilate major fermentation by-products generated by neighboring bacteria. Collectively, this suggests that *P. apium* metabolism in the queen ileum may be somewhat analogous to *S. alvi* function in the worker ileum [57].

Patterns of species co-occurrence suggest selection pressure for honey bee gut bacteria to co-exist with other bacteria in a biofilm encouraging competition and co-evolution (Fig. 4, Additional file 12: Table S12). This hypothesis is supported by the complex of highly correlated bacteria on the queen mouth and strongly affiliated species pairs occurring regardless of niche. Not strongly associated with age, niche, or background, at least three pairs of co-occurring species emerge as potential syntrophic relationships throughout the queen microbiota, and may rely on co-evolved traits to ensure niche occupation. This strategy would prove more effective in the queen gut, which provides a more stable long-term environment where partnerships have more generational time to evolve. Many bacterial pairs have evolved strict affiliations with one another and multiple hive niches including *P. apium/L. kunkeei*, *G apicola/S. alvi*, and *Lactobacillus* firm4*/B. asteroides* (Figs. 3 and 4). Perhaps through their reliance on one another, core bacteria better survive within and outside their preferred niche.

## Conclusions

The honey bee is a metabolic model for the effects of aging and diet on microbiota. Sampling the honey bee microbiota with respect to chronological age, biological age, and environmental exposure facilitates an informative partitioning of variation associated with longevity phenotypes. Consistent with established differences in host diet, development and aging, the queen microbiota shifts toward the fermentative metabolism of well-known gram-positive species, while the more rapidly aging worker is progressively depleted of these same species. Given the spectrum of influence of gut microbiota on worker physiology, we suggest that the queen microbiota serves a similarly critical role in host signaling and protection. Separate evolutionary trajectories for caste-specific gut bacteria reflect overt differences in diet and longevity between workers and queens. This trajectory appears to have tracked division of labor evolution, perhaps involving key innovations like nectar concentration to produce honey, and the production of royal jelly in worker hypopharyngeal glands. Once considered bacteria associated with worker gut dysbiosis and larval nutrition, *L. kunkeei* and *P. apium* must now be understood as core gut bacteria of *Apis mellifera* queens. Our results suggest that these two species occupy a functional niche in the queen mouth, midgut, and ileum. The co-occurrence and correlational abundance of multiple core species throughout the honey bee system suggest syntrophic relationships are commonplace. More generally, our study highlights the importance of controlled temporal and tissue-specific data to understand the total diversity and function of the honey bee microbiome.

## Additional files

Additional file 1: **Table S1.** Queen 16S gene read count, taxonomy and coding. (XLSX 427 kb)

Additional file 2: **Table S2.** Queen diversity, abundance, qPCR. (XLSX 484 kb)

Additional file 3: **Table S3** Queen relative and normalized abundance. (XLSX 462 kb)

Additional file 4: **Table S4.** MANOVA of queen age and source by niche. (XLSX 96 kb)

Additional file 5: **Table S5.** Wilcoxon rank sum test of queen age. (XLSX 12 kb)

Additional file 6: **Table S6.** MANOVA of worker gut microbiota by age. (XLSX 206 kb)

Additional file 7: **Table S7.** ANOVA of carbonyl by queen age and source. (XLSX 102 kb)

Additional file 8: **Table S8.** MANCOVA by queen age and source and carbonyl. (XLSX 145 kb)

Additional file 9: **Table S9.** Queen Pearsons correlations by carbonyl. (XLSX 11 kb)

Additional file 10: **Table S10.** PCA of queen microbial communities. (XLSX 222 kb)

Additional file 11: **Table S11.** Anosim by queen age and source. (XLSX 94 kb)

Additional file 12: **Table S12.** Queen microbiota correlations. (XLSX 3421 kb)

## Abbreviations

16S rRNA gene: 16S subunit of the ribosomal RNA gene
ANOVA: Analysis of variance
AZ: Arizona
BCA: Bicinchoninic acid
BLAST: Basic local alignment search tool
bp: Base pairs
CA: California
CLR: Centered log ratio
DistLM: Distance-based linear model
DNA: Deoxyribonucleic acid
DNPH: 2,4-Dinitrophenylhydrazine
FDR: False discovery rate
GLM: General linear models
MANCOVA: Multivariate analysis of covariance
MANOVA: Multivariate analysis of variance
OTU: Operational taxonomic unit
PCA: Principal components analysis
qPCR: Quantitative polymerase chain reaction
RDP: Ribosomal Database Project
rRNA: ribosomal ribonucleic acid
SparCC: Sparse correlations for compositional data algorithm

## References

[1] Seeley TD (1982). Adaptive significance of the age polyeithism schedule in honeybee colonies. Behav Ecol Sociobiol.

[2] Toth AL, Robinson GE (2005). Worker nutrition and division of labour in honeybees. Anim Behav.

[3] Korb J (2016). Why do social insect queens live so long? Approaches to unravel the sociality-aging puzzle. Curr Opin Insect Sci.

[4] Mao W, Schuler MA, Berenbaum MR (2015). A dietary phytochemical alters caste-associated gene expression in honey bees. Sci Adv.

[5] Kamakura M (2012). Royalactin induces queen differentiation in honeybees. Nature.

[6] Buttstedt A, Moritz RF, Erler S (2013). More than royal food—major royal jelly protein genes in sexuals and workers of the honeybee *Apis mellifera*. Front Zool.

[7] Vojvodic S, Rehan SM, Anderson KE (2013). Microbial gut diversity of Africanized and European honey bee larval instars. PLoS One.

[8] Haddad LS, Kelbert L, Hulbert AJ (2007). Extended longevity of queen honey bees compared to workers is associated with peroxidation-resistant membranes. Exp Gerontol.

[9] Corona M, Hughes KA, Weaver DB, Robinson GE (2005). Gene expression patterns associated with queen honey bee longevity. Mech Ageing Dev.

[10] Remolina SC, Hughes KA (2008). Evolution and mechanisms of long life and high fertility in queen honey bees. Age.

[11] Yang W, Tian Y, Han M, Miao X. Longevity extension of worker honey bees ( *Apis mellifera* ) by royal jelly: optimal dose and active ingredient. Peer J. 2017;5:e3118. 10.7717/peerj.3118 PMC5372980 28367370

[12] Anderson KE, Ricigliano VA (2017). Honey bee gut dysbiosis: a novel context of disease ecology. Curr. Opin. Insect Sci..

[13] Engel P, Kwong WK, Mcfrederick Q, Anderson KE, Barribeau M, Chandler JA (2016). The bee microbiome: impact on bee health and model for evolution and ecology of host-microbe interactions. mBIO.

[14] Amdam GV, Omholt SW (2002). The regulatory anatomy of honeybee lifespan. J Theor Biol.

[15] Amdam GV, Norberg K, Hagen A, Omholt SW (2003). Social exploitation of vitellogenin. Proc Natl Acad Sci.

[16] Corona M, Velarde RA, Remolina S, Moran-Lauter A, Wang Y, Hughes KA (2007). Vitellogenin, juvenile hormone, insulin signaling, and queen honey bee longevity. Proc Natl Acad Sci.

[17] Amdam GV, Simões ZLP, Hagen A, Norberg K, Schrøder K, Mikkelsen Ø (2004). Hormonal control of the yolk precursor vitellogenin regulates immune function and longevity in honeybees. Exp Gerontol.

[18] Seehuus S-C, Norberg K, Gimsa U, Krekling T, Amdam GV (2006). Reproductive protein protects functionally sterile honey bee workers from oxidative stress. Proc Natl Acad Sci.

[19] Kwong WK, Medina LA, Koch H, Sing K-W, Jia E, Soh Y (2017). Dynamic microbiome evolution in social bees. Sci. Adv.

[20] Tarpy DR, Mattila HR, Newton ILG (2015). Development of the honey bee gut microbiome throughout the queen-rearing process. Appl Environ Microbiol.

[21] Vojvodic S, Johnson BR, Harpur BA, Kent CF, Zayed A, Anderson KE (2015). The transcriptomic and evolutionary signature of social interactions regulating honey bee caste development. Ecol. Evol.

[22] Anderson KE, Rodrigues PAP, Mott BM, Maes P, Corby-Harris V (2016). Ecological succession in the honey bee gut: shift in *Lactobacillus* strain dominance during early adult development. Microb Ecol.

[23] Powell JE, Martinson VG, Urban-Mead K, Moran NA (2014). Routes of acquisition of the gut microbiota of the honey bee *Apis mellifera*. Appl Environ Microbiol.

[24] Ricigliano VA, Fitz W, Copeland DC, Mott BM, Maes P, Floyd AS (2017). The impact of pollen consumption on honey bee (*Apis mellifera*) digestive physiology and carbohydrate metabolism. Arch Insect Biochem Physiol.

[25] Zheng H, Powell JE, Steele MI, Dietrich C, Moran NA (2017). Honeybee gut microbiota promotes host weight gain via bacterial metabolism and hormonal signaling. Proc Natl Acad Sci.

[26] Powell JE, Leonard SP, Kwong WK, Engel P, Moran NA (2016). Genome-wide screen identifies host colonization determinants in a bacterial gut symbiont. Proc Natl Acad Sci.

[27] Kwong WK, Mancenido AL, Moran NA (2017). Immune system stimulation by the native gut microbiota of honey bees. R Soc Open Sci.

[28] Emery O, Schmidt K, Engel P (2017). Immune system stimulation by the gut symbiont *Frischella perrara* in the honey bee (*Apis mellifera*). Mol Ecol.

[29] Maes PW, Rodrigues PAP, Oliver R, Mott BM, Anderson KE (2016). Diet-related gut bacterial dysbiosis correlates with impaired development, increased mortality and *Nosema* disease in the honeybee (Apis mellifera). Mol Ecol.

[30] Buford TW (2017). (Dis)Trust your gut: the gut microbiome in age-related inflammation, health, and disease. Microbiome.

[31] Kapheim KM, Rao VD, Yeoman CJ, Wilson BA, White BA, Goldenfeld N (2015). Caste-specific differences in hindgut microbial communities of honey bees (*Apis mellifera*). PLoS One.

[32] Anderson KE, Carroll MJ, Sheehan TIM, Mott BM (2014). Hive-stored pollen of honey bees: many lines of evidence are consistent with pollen preservation, not nutrient conversion. Mol Ecol.

[33] Corby-Harris V, Snyder LA, Schwan MR, Maes P, McFrederick QS, Anderson KE (2014). Origin and effect of Alpha 2.2 Acetobacteraceae in honey bee larvae and description of *Parasaccharibacter apium* gen. nov., sp. nov. Appl Environ Microbiol.

[34] Cariveau DP, Powell JE, Koch H, Winfree R, Moran NA (2014). Variation in gut microbial communities and its association with pathogen infection in wild bumble bees (*Bombus*). ISME J.

[35] Djukic M, Poehlein A, Strauß J, Tann FJ, Leimbach A, Hoppert M (2015). High quality draft genome of *Lactobacillus kunkeei* EFB6, isolated from a German European foulbrood outbreak of honeybees. Stand Genomic Sci.

[36] Endo A, Salminen S (2013). Honeybees and beehives are rich sources for fructophilic lactic acid bacteria. Syst Appl Microbiol.

[37] Martinson VG, Moy J, Moran NA (2012). Establishment of characteristic gut bacteria during development of the honeybee worker. Appl Environ Microbiol.

[38] Hsieh Y-S, Hsu C-Y (2011). Honeybee trophocytes and fat cells as target cells for cellular senescence studies. Exp Gerontol.

[39] Smith PK, Krohn RI, Hermanson GT, Mallia AK, Gartner FH, Provenzano MD (1985). Measurement of protein using bicinchoninic acid. Anal Biochem.

[40] Liu CM, Aziz M, Kachur S, Hsueh P-R, Huang Y-T, Keim P (2012). BactQuant: an enhanced broad-coverage bacterial quantitative real-time PCR assay. BMC Microbiol.

[41] Schloss PD, Westcott SL, Ryabin T, Hall JR, Hartmann M, Hollister EB (2009). Introducing mothur: open-source, platform-independent, community-supported software for describing and comparing microbial communities. Appl Environ Microbiol.

[42] Edgar RC, Haas BJ, Clemente JC, Quince C, Knight R (2011). UCHIME improves sensitivity and speed of chimera detection. Bioinformatics.

[43] Wang Q, Garrity GM, Tiedje JM, Cole JR (2007). Naive Bayesian classifier for rapid assignment of rRNA sequences into the new bacterial taxonomy. Appl Environ Microbiol.

[44] Chouaia B, Gaiarsa S, Crotti E, Comandatore F, Esposti MD, Ricci I (2014). Acetic acid bacteria genomes reveal functional traits for adaptation to life in insect guts. Genome Biol. Evol.

[45] Větrovský T, Baldrian P (2013). The variability of the 16S rRNA gene in bacterial genomes and its consequences for bacterial community analyses. PLoS One.

[46] Pearson K (1896). Mathematical contributions to the theory of evolution—on a form of spurious correlation which may arise when indices are used in the measurement of organs. Proc R Soc London.

[47] Gloor GB, Reid G. Compositional analysis: a valid approach to analyze microbiome high throughput sequencing data. Can J Microbiol. 2016;703 cjm-2015-0821 10.1139/cjm-2015-0821 27314511

[48] Comas M, Thio-Henestrosa S. CoDaPack 2.0: a stand-alone, multi-platform compositional software. In: Egozcue JJ, Tolosana-Delgado R, Ortego MI, Editors. 4th International Workshop on Compositional Data Analysis. 2011:1–10.

[49] Friedman J, Alm EJ (2012). Inferring correlation networks from genomic survey data. PLoS Comput Biol.

[50] Weiss S, Van Treuren W, Lozupone C, Faust K, Friedman J, Deng Y (2016). Correlation detection strategies in microbial data sets vary widely in sensitivity and precision. Isme J.

[51] 2013 SAS Institute Inc (2013). Base SAS® 9.4 Procedures Guide.

[52] Salmela H, Sundström L. Vitellogenin in inflamation and immunity in social inects. Inflamm Cell. 2017;4:e1506.

[53] Amdam GV (2011). Social context, stress, and plasticity of aging. Aging Cell.

[54] Engel P, Bartlett K, Moran NA (2015). The bacterium *Frischella perrara* causes scab formation in the gut of its honey bee host. MBio.

[55] Bonilla-Rosso G, Engel P (2018). Functional roles and metabolic niches in the honey bee gut microbiota. Curr Opin Microbiol.

[56] Kwong WK, Engel P, Koch H, Moran NA (2014). Genomics and host specialization of honey bee and bumble bee gut symbionts. Proc Natl Acad Sci.

[57] Kwong WK, Moran NA (2016). Gut microbial communities of social bees. Nat Rev Microbiol.

[58] Moran NA, Hansen AK, Powell JE, Sabree ZL (2012). Distinctive gut microbiota of honey bees assessed using deep sampling from individual worker bees. PLoS One.

[59] Kešnerová L, Mars RAT, Ellegaard KM, Troilo M (2017). Disentangling metabolic functions of bacteria in the honey bee gut. PLoS Biol.

[60] Corby-Harris V, Maes P, Anderson KE (2014). The bacterial communities associated with honey bee (Apis mellifera) foragers. PLoS One.

[61] Horton MA, Oliver R, Newton IL (2015). No apparent correlation between honey bee forager gut microbiota and honey production. Peer J.

[62] Rothman JA, Carroll MJ, Meikle WG, Anderson KE, McFrederick QS. Longitudinal effects of supplemental forage on the honey bee (*Apis mellifera*) microbiota and inter- and intra-colony variability. Microb Ecol. 2018;75:1–11. 10.1007/s00248-018-1151-y 29397399

[63] Yun J-H, Jung M-J, Kim PS, Bae J-W (2018). Social status shapes the bacterial and fungal gut communities of the honey bee. Sci Rep.

[64] Jones JC, Fruciano C, Hildebrand F, Al Toufalilia H, J Balfour N, Bork P, et al. Gut microbiota composition is associated with environmental landscape in honey bees. Ecol Evol. 2017;8:441–451. 10.1002/ece3.3597 PMC5756847 29321884

[65] Keller L, Jemielity S (2006). Social insects as a model to study the molecular basis of ageing. Exp Gerontol.

[66] Amiri E, Strand MK, Rueppell O, Tarpy DR (2017). Queen quality and the impact of honey bee diseases on queen health: potential for interactions between two major threats to colony health. Insects.

[67] Rivière A, Selak M, Lantin D, Leroy F, De Vuyst L (2016). *Bifidobacteria* and butyrate-producing colon bacteria: importance and strategies for their stimulation in the human gut. Front Microbiol.

[68] Hu YT, Wu TC, Yang EC, Wu PC, Lin PT, Wu YL (2017). Regulation of genes related to immune signaling and detoxification in *Apis mellifera* by an inhibitor of histone deacetylation. Sci Rep.

[69] Heylen K, Gobin B, Billen J, Hu TT, Arckens L, Huybrechts R. Amfor expression in the honeybee brain: a trigger mechanism for nurse-forager transition. J Insect Physiol. 2008;54:1400–3. 10.1016/j.jinsphys.2008.07.015 18725227

[70] Suzuki MT, Giovannoni SJ. Bias caused by template annealing in the amplification of mixtures of 16S rRNA genes by PCR†. Appl Environ Microbiol. 1996;62:625–630. 10.1128/aem.62.2.625-630.1996 PMC167828 8593063

[71] Ward KN, Coleman JL, Clinnin K, Fahrbach S, Rueppell O (2008). Age, caste, and behavior determine the replicative activity of intestinal stem cells in honeybees (*Apis mellifera* L.). Exp Gerontol.

[72] vanEngelsdorp D, Tarpy DR, Lengerich EJ, Pettis JS (2013). Idiopathic brood disease syndrome and queen events as precursors of colony mortality in migratory beekeeping operations in the eastern United States. Prev Vet Med.

[73] Corby-Harris V, Snyder L, Meador CAD, Naldo R, Mott B, Anderson KE (2016). *Parasaccharibacter apium*, gen. Nov., sp. Nov., improves honey bee (Hymenoptera: Apidae) resistance to *Nosema*. J Econ Entomol.

[74] Alberoni D, Gaggìa F, Baffoni L, Di Gioia D (2016). Beneficial microorganisms for honey bees: problems and progresses. Appl Microbiol Biotechnol.

[75] McFrederick QS, Wcislo WT, Taylor DR, Ishak HD, Dowd SE, Mueller UG (2012). Environment or kin: whence do bees obtain acidophilic bacteria?. Mol Ecol.

[76] Graystock P, Rehan SM, McFrederick QS (2017). Hunting for healthy microbiomes: determining the core microbiomes of *Ceratina*, *Megalopta*, and *Apis* bees and how they associate with microbes in bee collected pollen. Conserv Genet.

[77] McFrederick QS, Thomas JM, Neff JL, Vuong HQ, Russell KA, Hale AR, et al. Flowers and wild megachilid bees share microbes. Microb Ecol. 2017;73:188–200. 10.1007/s00248-016-0838-1 27592345

[78] McFrederick QS, Wcislo WT, Hout MC, Mueller UG (2014). Host species and developmental stage, but not host social structure, affects bacterial community structure in socially polymorphic bees. FEMS Microbiol Ecol.

[79] McFrederick QS, Rehan SM (2016). Characterization of pollen and bacterial community composition in brood provisions of a small carpenter bee. Mol Ecol.

[80] Asenjo F, Olmos A, Henríquez-Piskulich P, Polanco V, Aldea P, Ugalde JA, et al. Genome sequencing and analysis of the first complete genome of *Lactobacillus kunkeei* strain MP2, an *Apis mellifera* gut isolate. Peer J. 2016;4:e1950. 10.7717/peerj.1950 PMC4841242 27114887

